# 4-Methyl­benzene­carbothio­amide

**DOI:** 10.1107/S1600536810015813

**Published:** 2010-05-08

**Authors:** Saqib Ali, Shahid Hameed, Ahmad Luqman, Tashfeen Akhtar, Masood Parvez

**Affiliations:** a Department of Chemistry, Quaid-i-Azam University, Islamabad 45320, Pakistan; b Department of Chemistry, The University of Calgary, 2500 University Drive NW, Calgary, Alberta, Canada T2N 1N4

## Abstract

In the title mol­ecule, C_8_H_9_NS, the mean plane of the carbothio­amide group is twisted slightly with respect to the mean plane of the benzene ring, making a dihedral angle of 17.03 (10)°. The crystal structure is stabilized by inter­molecular N—H⋯S hydrogen bonds, resulting in the formation of eight-membered rings lying about inversion centers and representing *R*_2_^2^(8) and *R*_4_^2^(8) motifs. Futhermore, these hydrogen bonds build up chains parallel to the *b* axis.

## Related literature

For the use of thio­amides as inter­mediates in the synthesis of various heterocyclic compounds, see: Zahid *et al.* (2009 ▶). For the uses of thio­amides, see: Lebana *et al.* (2008 ▶). For the biological activity of thio­amides, see: Jagodzinski (2003 ▶); Klimesova *et al.* (1999 ▶). For related structures, see: Khan *et al.* (2009*a* ▶,*b* ▶,*c* ▶); Jian *et al.* (2006 ▶); Ali *et al.* (2010 ▶). For graph-set notation, see: Etter *et al.* (1990 ▶); Bernstein *et al.* (1994 ▶).
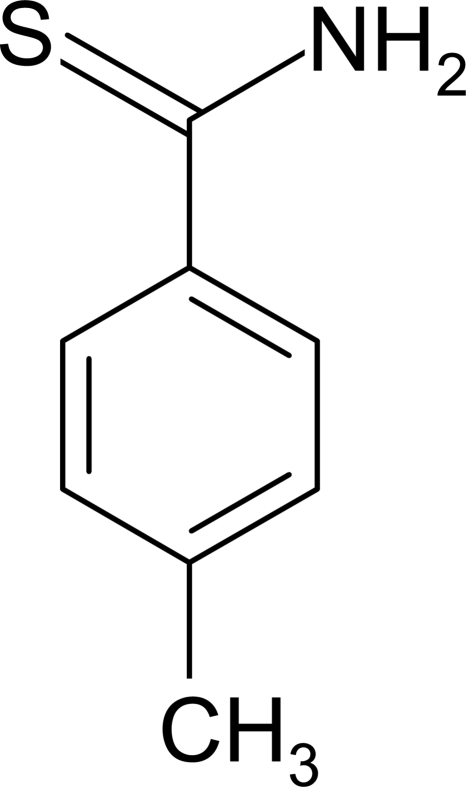


## Experimental

### 

#### Crystal data

C_8_H_9_NS
*M* *_r_* = 151.22
Monoclinic, 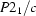

*a* = 9.7341 (5) Å
*b* = 5.8391 (2) Å
*c* = 13.9055 (6) Å
β = 104.946 (3)°
*V* = 763.63 (6) Å^3^
*Z* = 4
Mo *K*α radiation
μ = 0.34 mm^−1^
*T* = 123 K
0.10 × 0.06 × 0.06 mm

#### Data collection

Nonius KappaCCD diffractometer
Absorption correction: multi-scan (*SORTAV*; Blessing, 1997 ▶) *T*_min_ = 0.967, *T*_max_ = 0.980
2741 measured reflections
1482 independent reflections
1399 reflections with *I* > 2σ(*I*)
*R*_int_ = 0.025

#### Refinement

*R*[*F* ^2^ > 2σ(*F* ^2^)] = 0.034
*wR*(*F* ^2^) = 0.089
*S* = 1.06
1482 reflections
92 parameters
H-atom parameters constrained
Δρ_max_ = 0.27 e Å^−3^
Δρ_min_ = −0.24 e Å^−3^

### 

Data collection: *COLLECT* (Hooft, 1998 ▶); cell refinement: *DENZO* (Otwinowski & Minor, 1997 ▶); data reduction: *SCALEPACK* (Otwinowski & Minor, 1997 ▶); program(s) used to solve structure: *SHELXS97* (Sheldrick, 2008 ▶); program(s) used to refine structure: *SHELXL97* (Sheldrick, 2008 ▶); molecular graphics: *ORTEP-3 for Windows* (Farrugia, 1997 ▶); software used to prepare material for publication: *SHELXL97*.

## Supplementary Material

Crystal structure: contains datablocks global, I. DOI: 10.1107/S1600536810015813/dn2557sup1.cif

Structure factors: contains datablocks I. DOI: 10.1107/S1600536810015813/dn2557Isup2.hkl

Additional supplementary materials:  crystallographic information; 3D view; checkCIF report

## Figures and Tables

**Table 1:** Hydrogen-bond geometry (Å, °)

*D*—H⋯*A*	*D*—H	H⋯*A*	*D*⋯*A*	*D*—H⋯*A*
N1—H1*B*⋯S1^i^	0.88	2.56	3.4178 (14)	166
N1—H1*A*⋯S1^ii^	0.88	2.75	3.3179 (15)	124

Symmetry codes: (i) 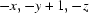; (ii) 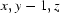.

**Fig. 1.**
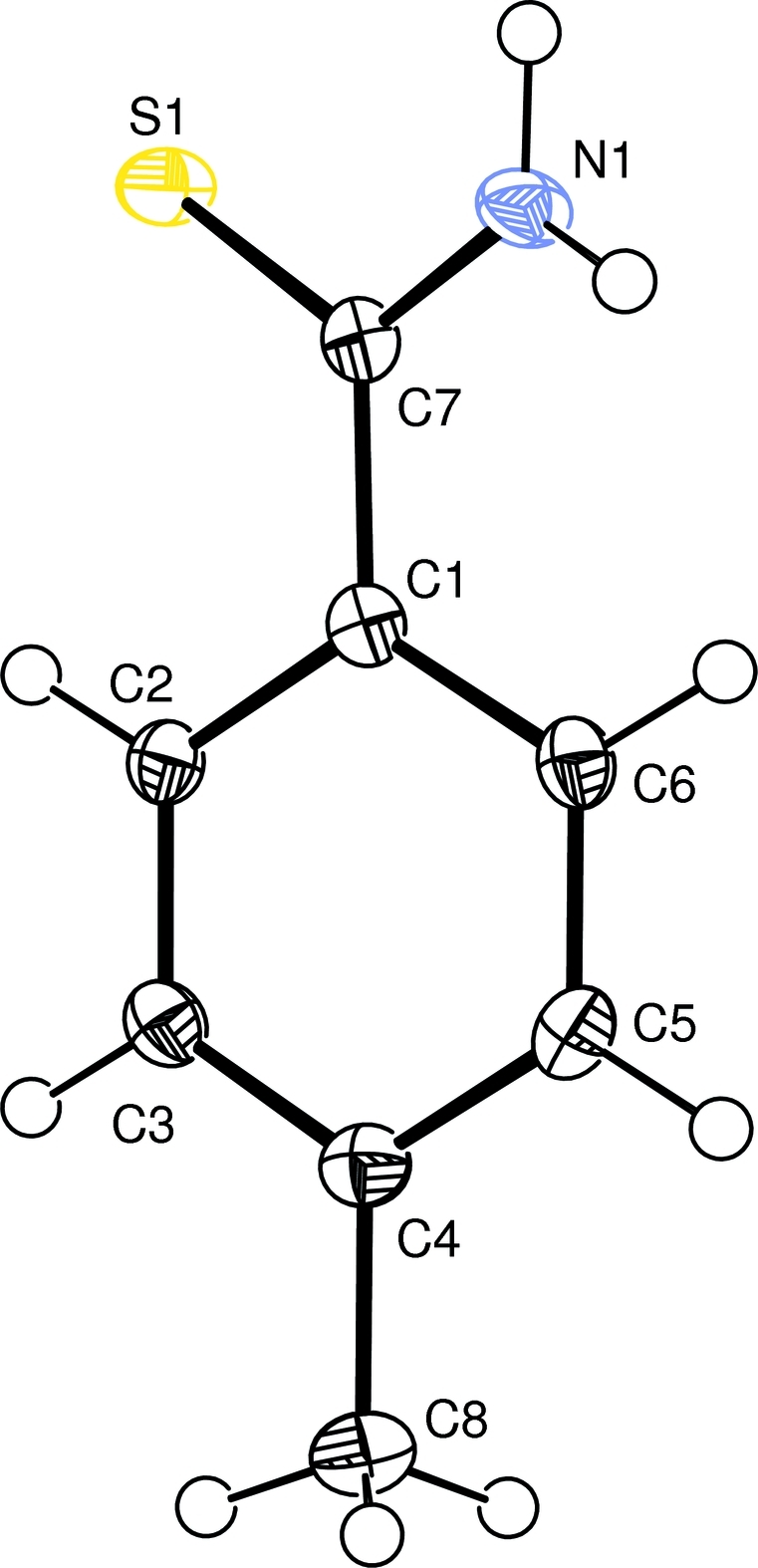
Molecular view of title compound with the atom labeling scheme. Ellipsoids are drawn at the 50% probability level. H atoms are represented as small sphere of arbitrary radii.

**Fig. 2.**
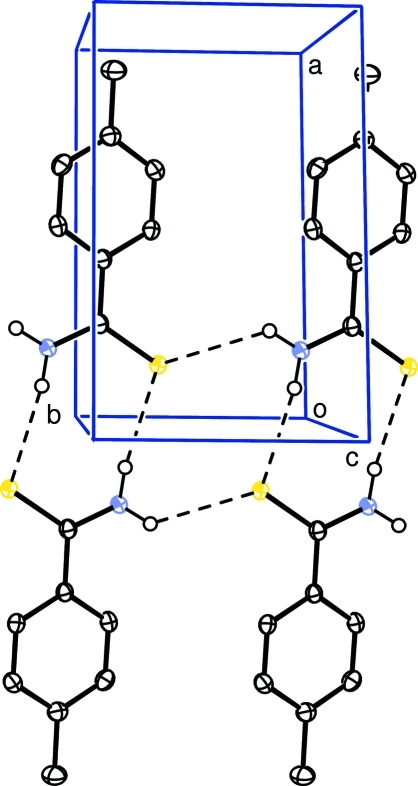
A part of the unit cell showing the N-H···S hydrogen bonds as dashed lines. H-atoms not involved in H-bonds have been excluded for clarity.

## supplementary crystallographic information

### Comment

Thioamides are not only used as intermediates in the synthesis of various heterocyclic compounds (Zahid *et al.*, 2009), they are important biologically active agents (Jagodzinski, 2003; Klimesova *et al.*, 1999). In addition, they are important ligands in the field of coordination chemistry (Lebana *et al.*, 2008). In continuation to our work on thioamides (Khan *et al.*, 2009*a*; 2009*b*; 2009*c*; Ali *et al.*, 2010), we have synthesized 4-methylbenzenecarbothioamide, (I). In this article we report the crystal structure of the title compound.

In the title molecule (Fig. 1), the bond distances and angles agree with the corresponding bond distances and angles reported in closely related compounds (Khan *et al.*, 2009*a*; 2009*b*; 2009*c*; Jian *et al.*, 2006; Ali *et al.*, 2010). In the title compound, the mean-plane of the carbothioamide group (S1/N1/C7) is slightly twisted with respect to the mean-plane of the phenyl ring (C1–C6), making a dihedral angle of 17.03 (10)°.

The structure is stabilized by intermolecular N—H···S hydrogen bonds resulting in the formation of eight membered rings lying about inversion centers (Tab. 1 and Fig. 2). In the graph set notation (Etter *et al.*, 1990; Bernstein *et al.*, 1994) the hydrogen bonded rings may be best described as representing R_2_^2^(8) and R_4_^2^(8) motifs.Futhermore, these hydrogen bonds build up chains parallel to the b axis.

### Experimental

4-Methylbenzonitrile (13.2 mmol) was added to a slurry of magnesium cholride hexahydrate (13.2 mmol) and sodium hydrogen sulphide hydrate (70%, 26.4 mmol) in dimethylformamide (35 ml) and the reaction mixture was stirred at room temperature for 4 h. The reaction mixture was poured into water (100 ml) and the resulting precipitates were collected by filtration. The product obtained was resuspended in 1 N HCl (50 ml), stirred for another 25 min, the precipitated solid filtered and washed with water. Recrystallization of the product from chloroform afforded the crystals of the title compound suitable for X-ray analysis.

### Refinement

Though all the H atoms could be distinguished in the difference Fourier map the H-atoms were included at geometrically idealized positions and refined in riding-model approximation with N—H = 0.88 Å and C—H = 0.95 and 0.98 Å for aryl and methyl H-atoms, respectively. The *U*_iso_(H) were allowed at 1.2/1.5*U*_eq_(N/C). The final difference map was essentially featurless.

### Crystal data

**Table:**

C_8_H_9_NS	*F*(000) = 320
*M**_r_* = 151.22	*D*_x_ = 1.315 Mg m^−^^3^
Monoclinic, *P*2_1_/*c*	Mo *K*α radiation, λ = 0.71073 Å
Hall symbol: -P 2ybc	Cell parameters from 1473 reflections
*a* = 9.7341 (5) Å	θ = 1.0–26.0°
*b* = 5.8391 (2) Å	µ = 0.34 mm^−^^1^
*c* = 13.9055 (6) Å	*T* = 123 K
β = 104.946 (3)°	Block, yellow
*V* = 763.63 (6) Å^3^	0.10 × 0.06 × 0.06 mm
*Z* = 4	

### Data collection

**Table:**

Nonius KappaCCD diffractometer	1482 independent reflections
Radiation source: fine-focus sealed tube	1399 reflections with *I* > 2σ(*I*)
graphite	*R*_int_ = 0.025
ω and φ scans	θ_max_ = 26.0°, θ_min_ = 3.8°
Absorption correction: multi-scan (*SORTAV*; Blessing, 1997)	*h* = −11→11
*T*_min_ = 0.967, *T*_max_ = 0.980	*k* = −7→7
2741 measured reflections	*l* = −16→16

### Refinement

**Table:**

Refinement on *F*^2^	Primary atom site location: structure-invariant direct methods
Least-squares matrix: full	Secondary atom site location: difference Fourier map
*R*[*F*^2^ > 2σ(*F*^2^)] = 0.034	Hydrogen site location: difference Fourier map
*wR*(*F*^2^) = 0.089	H-atom parameters constrained
*S* = 1.06	*w* = 1/[σ^2^(*F*_o_^2^) + (0.0373*P*)^2^ + 0.5912*P*] where *P* = (*F*_o_^2^ + 2*F*_c_^2^)/3
1482 reflections	(Δ/σ)_max_ < 0.001
92 parameters	Δρ_max_ = 0.27 e Å^−^^3^
0 restraints	Δρ_min_ = −0.24 e Å^−^^3^

### Special details

**Table:**

Geometry. All esds (except the esd in the dihedral angle between two l.s. planes) are estimated using the full covariance matrix. The cell esds are taken into account individually in the estimation of esds in distances, angles and torsion angles; correlations between esds in cell parameters are only used when they are defined by crystal symmetry. An approximate (isotropic) treatment of cell esds is used for estimating esds involving l.s. planes.

### Fractional atomic coordinates and isotropic or equivalent isotropic displacement parameters (Å^2^)

**Table:**

	*x*	*y*	*z*	*U*_iso_*/*U*_eq_	
S1	0.15464 (4)	0.79266 (7)	0.03136 (3)	0.02316 (16)	
N1	0.19369 (15)	0.3538 (2)	0.06514 (10)	0.0219 (3)	
H1A	0.2463	0.2313	0.0844	0.026*	
H1B	0.1007	0.3410	0.0440	0.026*	
C1	0.41221 (16)	0.5673 (3)	0.10461 (11)	0.0168 (3)	
C2	0.48580 (17)	0.7599 (3)	0.08529 (11)	0.0189 (3)	
H2	0.4344	0.8849	0.0496	0.023*	
C3	0.63277 (18)	0.7714 (3)	0.11743 (12)	0.0207 (4)	
H3	0.6805	0.9045	0.1037	0.025*	
C4	0.71154 (17)	0.5908 (3)	0.16965 (11)	0.0203 (4)	
C5	0.63795 (18)	0.3995 (3)	0.19021 (11)	0.0211 (4)	
H5	0.6897	0.2753	0.2264	0.025*	
C6	0.49105 (17)	0.3872 (3)	0.15894 (11)	0.0196 (3)	
H6	0.4433	0.2557	0.1744	0.023*	
C7	0.25443 (17)	0.5571 (3)	0.06803 (11)	0.0177 (3)	
C8	0.87114 (18)	0.6011 (3)	0.20278 (13)	0.0280 (4)	
H8A	0.9033	0.7532	0.1883	0.042*	
H8B	0.9114	0.4848	0.1671	0.042*	
H8C	0.9028	0.5719	0.2745	0.042*	

### Atomic displacement parameters (Å^2^)

**Table:**

	*U*^11^	*U*^22^	*U*^33^	*U*^12^	*U*^13^	*U*^23^
S1	0.0174 (2)	0.0146 (2)	0.0353 (3)	0.00051 (14)	0.00272 (18)	0.00029 (16)
N1	0.0160 (7)	0.0155 (7)	0.0332 (8)	−0.0010 (6)	0.0045 (6)	0.0019 (6)
C1	0.0192 (8)	0.0154 (8)	0.0163 (7)	−0.0005 (6)	0.0055 (6)	−0.0017 (6)
C2	0.0205 (8)	0.0156 (7)	0.0204 (7)	0.0009 (6)	0.0050 (6)	0.0019 (6)
C3	0.0215 (8)	0.0192 (8)	0.0221 (8)	−0.0032 (6)	0.0066 (6)	−0.0004 (6)
C4	0.0193 (8)	0.0229 (8)	0.0182 (7)	0.0001 (6)	0.0041 (6)	−0.0033 (6)
C5	0.0240 (8)	0.0203 (8)	0.0180 (7)	0.0042 (6)	0.0037 (6)	0.0024 (6)
C6	0.0234 (8)	0.0158 (8)	0.0202 (7)	−0.0018 (6)	0.0068 (6)	0.0009 (6)
C7	0.0204 (8)	0.0161 (8)	0.0170 (7)	−0.0007 (6)	0.0057 (6)	−0.0007 (6)
C8	0.0194 (9)	0.0328 (10)	0.0299 (9)	0.0000 (7)	0.0026 (7)	0.0007 (8)

### Geometric parameters (Å, °)

**Table:**

S1—C7	1.6852 (16)	C3—H3	0.9500
N1—C7	1.322 (2)	C4—C5	1.396 (2)
N1—H1A	0.8800	C4—C8	1.503 (2)
N1—H1B	0.8800	C5—C6	1.385 (2)
C1—C2	1.396 (2)	C5—H5	0.9500
C1—C6	1.403 (2)	C6—H6	0.9500
C1—C7	1.489 (2)	C8—H8A	0.9800
C2—C3	1.386 (2)	C8—H8B	0.9800
C2—H2	0.9500	C8—H8C	0.9800
C3—C4	1.393 (2)		
			
C7—N1—H1A	120.0	C6—C5—C4	121.35 (15)
C7—N1—H1B	120.0	C6—C5—H5	119.3
H1A—N1—H1B	120.0	C4—C5—H5	119.3
C2—C1—C6	118.11 (15)	C5—C6—C1	120.48 (15)
C2—C1—C7	120.12 (14)	C5—C6—H6	119.8
C6—C1—C7	121.77 (14)	C1—C6—H6	119.8
C3—C2—C1	121.02 (15)	N1—C7—C1	117.39 (14)
C3—C2—H2	119.5	N1—C7—S1	120.35 (12)
C1—C2—H2	119.5	C1—C7—S1	122.26 (12)
C2—C3—C4	120.99 (15)	C4—C8—H8A	109.5
C2—C3—H3	119.5	C4—C8—H8B	109.5
C4—C3—H3	119.5	H8A—C8—H8B	109.5
C3—C4—C5	118.04 (15)	C4—C8—H8C	109.5
C3—C4—C8	121.05 (15)	H8A—C8—H8C	109.5
C5—C4—C8	120.91 (15)	H8B—C8—H8C	109.5
			
C6—C1—C2—C3	1.0 (2)	C4—C5—C6—C1	0.6 (2)
C7—C1—C2—C3	−179.07 (14)	C2—C1—C6—C5	−1.4 (2)
C1—C2—C3—C4	0.3 (2)	C7—C1—C6—C5	178.65 (14)
C2—C3—C4—C5	−1.1 (2)	C2—C1—C7—N1	162.91 (15)
C2—C3—C4—C8	178.76 (15)	C6—C1—C7—N1	−17.2 (2)
C3—C4—C5—C6	0.7 (2)	C2—C1—C7—S1	−17.1 (2)
C8—C4—C5—C6	−179.18 (15)	C6—C1—C7—S1	162.78 (12)

### Hydrogen-bond geometry (Å, °)

**Table:**

*D*—H···*A*	*D*—H	H···*A*	*D*···*A*	*D*—H···*A*
N1—H1B···S1^i^	0.88	2.56	3.4178 (14)	166
N1—H1A···S1^ii^	0.88	2.75	3.3179 (15)	124

Symmetry codes: (i) −*x*, −*y*+1, −*z*; (ii) *x*, *y*−1, *z*.
